# Interaction between gemcitabine and topotecan in human non-small-cell lung cancer cells: effects on cell survival, cell cycle and pharmacogenetic profile

**DOI:** 10.1038/sj.bjc.6602382

**Published:** 2005-02-08

**Authors:** E Giovannetti, V Mey, R Danesi, F Basolo, S Barachini, M Deri, M Del Tacca

**Affiliations:** 1 Division of Pharmacology and Chemotherapy, Department of Oncology, Transplants and Advanced Technologies in Medicine, University of Pisa, 55, Via Roma, 56126 Pisa, Italy; 2 Division of Pathological Anatomy and Histology, Department of Oncology, Transplants and Advanced Technologies in Medicine, University of Pisa, Pisa, Italy

**Keywords:** NSCLC cells, drug interaction, apoptosis, PI3-Akt, inducible gene expression

## Abstract

The pyrimidine analogue gemcitabine is an established effective agent in the treatment of non-small-cell lung cancer (NSCLC). The present study investigates whether gemcitabine would be synergistic with the topoisomerase I inhibitor topotecan against the NSCLC A549 and Calu-6 cells. Cells were treated with gemcitabine and topotecan for 1 h and the type of drug interaction was assessed using the combination index (CI). Cell cycle alterations were analysed by flow cytometry, while apoptosis was examined by the occurrence of DNA internucleosomal fragmentation, nuclear condensation and caspase-3 activation. Moreover, the possible involvement of the PI3K-Akt signalling pathway was investigated by the measurement of Akt phosphorylation. Finally, quantitative, real-time PCR (QRT-PCR) was used to study modulation of the gemcitabine-activating enzyme deoxycytidine kinase (dCK) and the cellular target enzyme ribonucleotide reductase (RR). In results, it was found that simultaneous and sequential topotecan → gemcitabine treatments were synergistic, while the reverse sequence was antagonistic in both cell lines. DNA fragmentation, nuclear condensation and enhanced caspase-3 activity demonstrated that the drug combination markedly increased apoptosis in comparison with either single agent, while cell cycle analysis showed that topotecan increased cells in S phase. Furthermore, topotecan treatment significantly decreased the amount of the activated form of Akt, and enhanced the expression of *dCK* (+155.0 and +115.3% in A549 and Calu-6 cells, respectively), potentially facilitating gemcitabine activity. In conclusion, these results indicate that the combination of gemcitabine and topotecan displays schedule-dependent activity *in vitro* against NSCLC cells. The gemcitabine → topotecan sequence is antagonistic while drug synergism is obtained with the simultaneous and the sequential topotecan → gemcitabine combinations, which are associated with induction of decreased Akt phosphorylation and increased *dCK* expression.

Despite recent advances in early diagnosis and treatment, non-small-cell lung cancer (NSCLC) is a disease with severe prognosis. The majority of patients with NSCLC are administered chemotherapy (Bunn and Kelly, 1998; Arriagada *et al*, 2004); however, the efficacy of cytotoxic drugs is still unsatisfactory, with a low percentage of complete remissions and short duration of clinical response. The NSCLC Collaborative Group (1995) meta-analysis study revealed a survival benefit of 10% at 1 year in a supportive care setting and an increased median survival of 6 weeks in patients treated with platinum-based chemotherapy, thus emphasising the need for new effective drugs and combination regimens. Clinical studies have shown that a number of new chemotherapeutic agents, such as gemcitabine, topoisomerase I inhibitors, taxanes and vinca alkaloids, are active agents in NSCLC (Bunn and Kelly, 1998). Preclinical studies with gemcitabine and etoposide (van Moorsel *et al*, 1999a, 1999b), gemcitabine and carboplatin or paclitaxel (Edelman *et al*, 2001), and topotecan and cisplatin (Kaufmann *et al*, 1996) have shown schedule-dependent drug interactions in several human lung cancer cell lines.

Results from clinical studies suggested that the combination of gemcitabine and topotecan compared favourably with others regimens accepted as reasonable treatment for NSCLC, and that further investigations of this combination is recommended (Dowlati and Levitan, 2003). A phase II trial of topotecan and gemcitabine in 35 patients with previously treated advanced NSCLC, of whom 17 had not responded to first-line therapy, demonstrated antitumour activity, with 11% partial responses and 23% stable diseases, a 1-year survival rate of 20% and a manageable toxicity profile (Rinaldi *et al*, 2002). In another phase I/II trial, based on preclinical data showing synergism against A549 cells, the combination of topotecan and gemcitabine was administered to 24 patients with previously untreated metastatic or unresectable NSCLC, and seemed to be active, with 21% partial responses and acceptable haematologic toxicity (Dabrow *et al*, 2003). Finally, a phase II multicentre study of combined topotecan and gemcitabine as first-line chemotherapy in 51 patients with advanced NSCLC showed a 1-year survival (39%) similar to platinum-based regimens, with 17% partial responses, a high percentage of patients achieving stable disease (23%) and no significant toxicities (Joppert *et al*, 2003).

Gemcitabine (dFdC, 2′,2′-difluoro-2′-deoxycytidine) is a deoxycytidine analogue with a broad spectrum of anticancer activity against several solid tumours in preclinical models (van Moorsel *et al*, 2000), and it is now an established effective agent in the treatment of NSCLC and pancreatic cancer (Noble and Goa, 1997). Gemcitabine is assumed to exert its antitumour effect mainly by incorporation of its triphosphate metabolite (dFdCTP) into DNA, after which DNA polymerase adds one additional deoxynucleotide and DNA synthesis is interrupted. The rate-limiting step in the activation of the drug is catalysed by deoxycytidine kinase (dCK), which is a limiting factor for the cytotoxic activity of gemcitabine. Furthermore, the diphosphate metabolite (dFdCDP) inhibits ribonucleotide reductase (RR), an enzyme that converts ribonucleotides to deoxyribonucleotides, required for DNA polymerisation and repair. Ribonucleotide reductase consists of dimerised large and small subunits 1 and 2 (RRM1 and RRM2), whose pairing is essential for scheduled DNA synthesis to occur. The interaction between gemcitabine and RR has not been well characterised; however, data support the hypothesis that the RRM1 subunit is the most important intracellular target of dFdCDP (van der Donk *et al*, 1998), although resistance to gemcitabine was observed both in RRM1- and RRM2-overexpressing cells (Goan *et al*, 1999; Davidson *et al*, 2004). Thus, gemcitabine resistance may be dependent on decreased expression of *dCK*, or overexpression of *RR*.

Topotecan is a water-soluble camptothecin derivative, which forms a stable, cleavable complex with the DNA-topoisomerase I. This process leads to breaks in the DNA strand resulting in apoptosis and cell death (Kollmannsberger *et al*, 1999). Recent studies demonstrated that topotecan exhibited its cytotoxic effects also by downregulating the PI3K-Akt signalling pathway, which is involved in prevention of apoptosis (Nakashio *et al*, 2002).

Gemcitabine and topotecan have different mechanisms of action, partially nonoverlapping toxicities and both drugs are active on tumour cells in the S-phase of the cell cycle (Tolis *et al*, 1999). Furthermore, the anti-topoisomerase I activity of topotecan might impair the excision-repair mechanisms of DNA and could potentiate the activity of gemcitabine, mainly by enhancement of apoptosis (Nakashio *et al*, 2000). Finally, selection of drugs to be combined with gemcitabine should be performed on the basis of their ability to modulate gene expression of critical enzymes *dCK* and *RR* (Bergman *et al*, 2002).

The purpose of the present study was to assess the efficacy of gemcitabine and topotecan combinations against NSCLC *in vitro*, studying several mechanisms involved in drug interaction, in order to provide experimental data in support of their clinical use in NSCLC.

## MATERIALS AND METHODS

### Drugs and chemicals

Topotecan and gemcitabine were generous gifts from GlaxoSmithKline (King of Prussia, PA, USA) and Eli Lilly Company (Indianapolis, IN, USA), respectively. Drugs were dissolved in sterile distilled water and diluted in culture medium immediately before use.

RPMI medium, fetal bovine serum (FBS), L-glutamine (2 mM), penicillin (50 IU ml^−1^), streptomycin (50 *μ*g ml^−1^), agarose and DNA ladder were from Gibco (Gaithersburg, MD, USA). All other chemicals were from Sigma Chemical Co. (St Louis, MO, USA).

### Cell culture

The NSCLC cell lines A549 (adenocarcinoma) and Calu-6 (epidermoid carcinoma) were from ATCC (Manassas, VA, USA) and were cultured, respectively, in RPMI and MEM (90%), supplemented with FBS (10%), L-glutamine (1%) and penicillin–streptomycin (1%), in an atmosphere of 5% CO_2_ at 37°C. Cells were routinely grown in 75 cm^2^ tissue culture flasks (Costar, Cambridge, MA, USA) and were harvested with a solution of trypsin–EDTA when they were in logarithmic phase of growth, and maintained at the above-described culture conditions for all experiments.

### Assay of cytotoxicity

Cells were harvested from cultures and plated in six-well sterile plastic plates (Costar, Cambridge, MA, USA) at 10^4^ cells well^−1^, and allowed to attach for 24 h. Cells were treated as follows: (1) gemcitabine (0.1–10^5^ ng ml^−1^) for 1 h; (2) topotecan (0.1–10^5^ ng ml^−1^) for 1 h; (3) topotecan for 1 h followed by medium change and then gemcitabine for 1 additional hour; (4) the reverse sequence of point (3) above and (5) simultaneous treatment (topotecan and gemcitabine for 1 h). After treatment, cells were cultured in drug-free medium and the growth inhibition by drugs was assessed by counting cells surviving after 72 h. Cell cytotoxicity was expressed as the percentage of control cell number, and the 50% inhibitory concentration of cell growth (IC_50_) was calculated by nonlinear least-squares curve fitting (GraphPad PRISM; Intuitive Software for Science, San Diego, CA, USA).

The type of drug interaction between gemcitabine and topotecan was assessed at a fixed concentration ratio of topotecan : gemcitabine, by using the combination index (CI) of Chou *et al* (1994). The data were processed by the Calcusyn software (Biosoft; Oxford, UK).

### Cell cycle analysis

Cell cycle alterations induced by treatments were studied by flow cytometry analysis. Cells were plated at a density of 1 × 10^6^ in 100-mm Petri dishes (Costar, Cambridge, MA, USA) and allowed to attach for 24 h. After single drug treatments with gemcitabine or topotecan at IC_50_ concentration levels, cells were collected by trypsinisation and washed twice with PBS. DNA staining was performed with a solution containing RNase (1 mg ml^−1^), Nonidet (0.1%) and propidium iodide (25 *μ*g ml^−1^) and the samples were stored on ice for 30 min. Analysis was performed using a FACScan (Becton Dickinson, San Jose, CA, USA) and data analysis was carried out with CELLQuest software, while cell cycle distribution was determined using Modfit software (Verity Software House, Inc., Topsham, ME, USA).

### Analysis of apoptosis by DNA fragmentation

Tumour cells (2 × 10^6^) were plated in 100 mm sterile dishes and treated with IC_50_ concentrations of drugs for 1 h. At the end of treatment, detection of apoptosis was performed as described previously (Danesi *et al*, 1996). Cells were lysed and treated with proteinase K. The DNA was precipitated with NaCl, ethanol and glycogen, dried, resuspended in Tris/EDTA, containing bovine pancreatic ribonuclease A and electrophoresed on a 1% agarose gel at 90 mV for 60 min. Bands were visualised using ethidium bromide staining, compared against a 180 DNA ladder for fragment size identification, and gels were photographed with a Polaroid MP4 Land Camera (Polaroid, Cambridge, MA, USA). Film densities of apoptosis assays were quantified through video imaging densitometry with the KS300 version 1.2 software (Kontron Elektronic, Eching, Germany).

### Analysis of apoptosis by nuclear staining

Gemcitabine, topotecan and their combinations were also characterised for their ability to induce nuclear condensation and fragmentation, as detected by bisbenzimide staining. Briefly, cells were treated as described above for DNA fragmentation analysis; at the end of incubation, cells were washed twice with PBS, fixed in 4% neutral-buffered paraformaldehyde, and incubated at room temperature for 15 min. Cells were then resuspended in a solution containing bisbenzimide trihydrochloride 8 *μ*g ml^−1^ and incubated for 15 min at room temperature. The cell suspensions were spotted on sylanised microscope glass slides and examined by fluorescence microscopy (Leica, Germany) for the presence of chromatin condensation and nuclear fragmentation. A total of 200 cells from randomly chosen microscopic fields were counted in a blinded fashion, and the apoptotic index was calculated as the percentage ratio between the number of cells displaying apoptotic appearance and the total number of counted cells.

### Assay of caspase-3 activity

To assess whether caspase-3 is activated upon treatment with gemcitabine or topotecan, enzyme activity was measured by the caspase-3 assay kit (Calbiochem, Oxford, UK). Cells were treated as described above for apoptosis analysis. At the end of incubation, cells were washed twice with PBS, harvested by centrifugation (1000 **g** for 10 min at 4°C), and resuspended in 50 *μ*l of lysis buffer (HEPES 50 nM, DTT 10 mM, EDTA 0.1 mM, CHAPS 0.1% and Nonidet P-40 0.1%) at 4°C for 15 min. The cell lysate was centrifuged at 10 000 **g** for 10 min at 4°C, and the supernatants, representing cytoplasmatic extracts, were transferred to a microtitre plate and mixed with assay buffer (HEPES 50 nM, DTT 10 mM, glycerol 10% and CHAPS 0.1%, pH 7.4) added to each well. The reaction was started by adding 10 *μ*l of caspase-3 colorimetric substrate (Ac-DEVD-pNA); controls were obtained with the caspase-3 inhibitor Ac-DEVD-CHO, to measure nonspecific hydrolysis of the substrate, and with the human recombinant caspase-3, to compare the activity of a known amount of enzyme with the caspase-3 activity in cells extracts. Plates were then incubated at 37°C for 10 min, after which the absorbance was read at 405 nm. A microtitre-plate reader conversion factor was then calculated, taking into account the concentration of *p*-nitroaniline in the calibration standard, and the activity of caspase-3 (pmol substrate min^−1^) in the samples was obtained as the slope of the curve × conversion factor. Finally, enzyme activity was normalised with respect to total protein content of each cell extract, as measured with the Lowry reagent, and expressed as pmol min^−1^ mg protein^−1^.

### Assay of Akt phosphorylation

**Figure 1:**
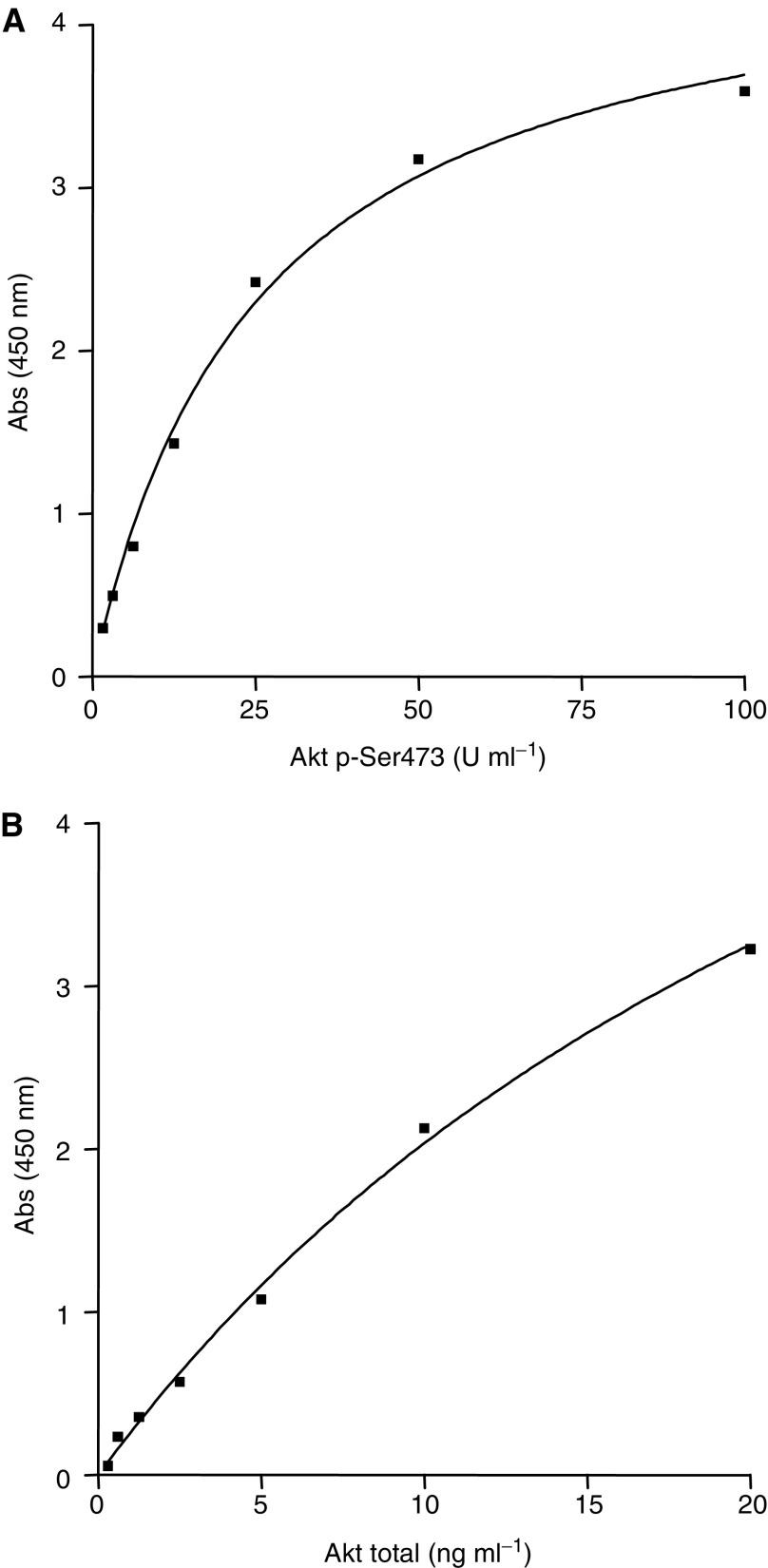
P-Ser473 Akt and Akt total standard curves for validation of P-Ser473-Akt activity ELISA assays. The following equations apply to the P-Ser473 Akt and Akt total concentrations: *y*=4.628*x*/(25.39+*x*), *R*^2^=0.99 (P-Ser473 Akt); and *y*=8.116*x*/(29.91+*x*), *R*^2^=0.99 (Akt total).

Akt protein activation by phosphorylation after gemcitabine or topotecan treatment was assayed with a P-Ser473-specific ELISA (BioSource International, Camarillo, CA, USA) and normalised to the total Akt content (BioSource). Cells were treated as described above for apoptosis analysis. At the end of incubation, cells were washed twice with PBS, harvested by centrifugation (1000 **g** for 5 min at 4°C), and resuspended in 25 *μ*l of extraction buffer (BioSource) for 30 min on ice, while vortexing as per protocol directions. A volume of 5 *μ*l of cell extract was diluted to 100 *μ*l in sodium azide (NaN_3_ 15 mM), centrifuged at 15 000 **g** for 10 min at 4°C and transferred to ELISA microtitre wells, coated with a monoclonal antibody specific for total Akt. A standard curve was run with each assay using 100, 50, 25, 12.5, 6.25, 3.12 and 1.6 U ml^−1^ of phosphorylated full-length human recombinant Akt (Akt P-Ser473) and 20, 10, 5, 2.5, 1.25, 0.6 and 0.3 ng ml^−1^ of human recombinant total Akt. After overnight incubation at 4°C, the solution was aspirated from wells and 100 *μ*l of rabbit anti-Akt P-Ser473 and biotin-conjugated anti-total Akt were added into each well of Akt P-Ser473 and total Akt, respectively. Plates were incubated at room temperature for 1 h, washed four times and 100 *μ*l of a working solution of a horseradish peroxidase-labelled anti-rabbit IgG and horseradish peroxidase-labelled streptavidin was added into each well of Akt P-Ser473 and total Akt ELISA assay, respectively. After 30 min, a chromogen solution was added; 20 min later, the reactions were stopped with 100 *μ*l of a stop solution and the absorbance was read at 450 nm. To calculate Akt P-Ser473 and total Akt concentrations, a standard curve method was used (Figure 1). Values of P-Ser473 calculated from the standard curve were then normalised for total Akt and protein contents.

### Quantitative, real-time PCR analysis

In order to assess the effect of treatments on expression of the key enzymes *dCK* and *RR*, total cellular RNA was extracted from cells treated as described above for cell cycle analysis, using TRI REAGENT LS (Sigma). RNA was dissolved in 10 mmol l^−1^ DTT and 200 U ml^−1^ RNase inhibitor in RNase free-water, and measured at 260 nm. RNA (1 *μ*g) was reverse transcribed at 37°C for 1 h in a 100-*μ*l reaction volume containing 0.8 mM deoxynucleotide mix (dNTPs), 200 U of Moloney murine leukemia virus reverse transcriptase (MMLV-RT), 40 U of RNase inhibitor and 0.05 *μ*g ml^−1^ random primers. The resulting cDNA was diluited (2 : 3) and then amplified by QRT-PCR with the Applied Biosystems 7900HT sequence detection system (Applied Biosystems, Foster City, CA, USA). Quantitative, real-time PCR reactions were performed in triplicate using 5 *μ*l of cDNA, 12.5 *μ*l of TaqMan Universal PCR Master Mix, 2.5 *μ*l of the specific probe and 2.5 *μ*l of the forward and reverse specific primers in a total volume of 25 *μ*l. PCR thermal cycling conditions, design and optimisation of primer concentrations were reported in detail by Giovannetti *et al* (2004). Amplifications were normalised to glyceraldehyde 3-phosphate dehydrogenase (GAPDH), and the quantitation of gene expression was performed using the ΔΔ*C*_t_ calculation, where *C*_t_ is the threshold cycle; the amount of target, normalised to the endogenous control and relative to the calibrator (untreated control cells), is given as 2^-ΔΔ*C*_t_^.

### Statistical analysis

All experiments were performed in triplicate and repeated at least three times. Data were expressed as mean values±s.e., and were analysed by Student's *t*-test or ANOVA followed by the Tukey's multiple comparison; the level of significance was set at *P*<0.05.

## RESULTS

### Cytotoxicity of gemcitabine and topotecan

**Figure 2:**
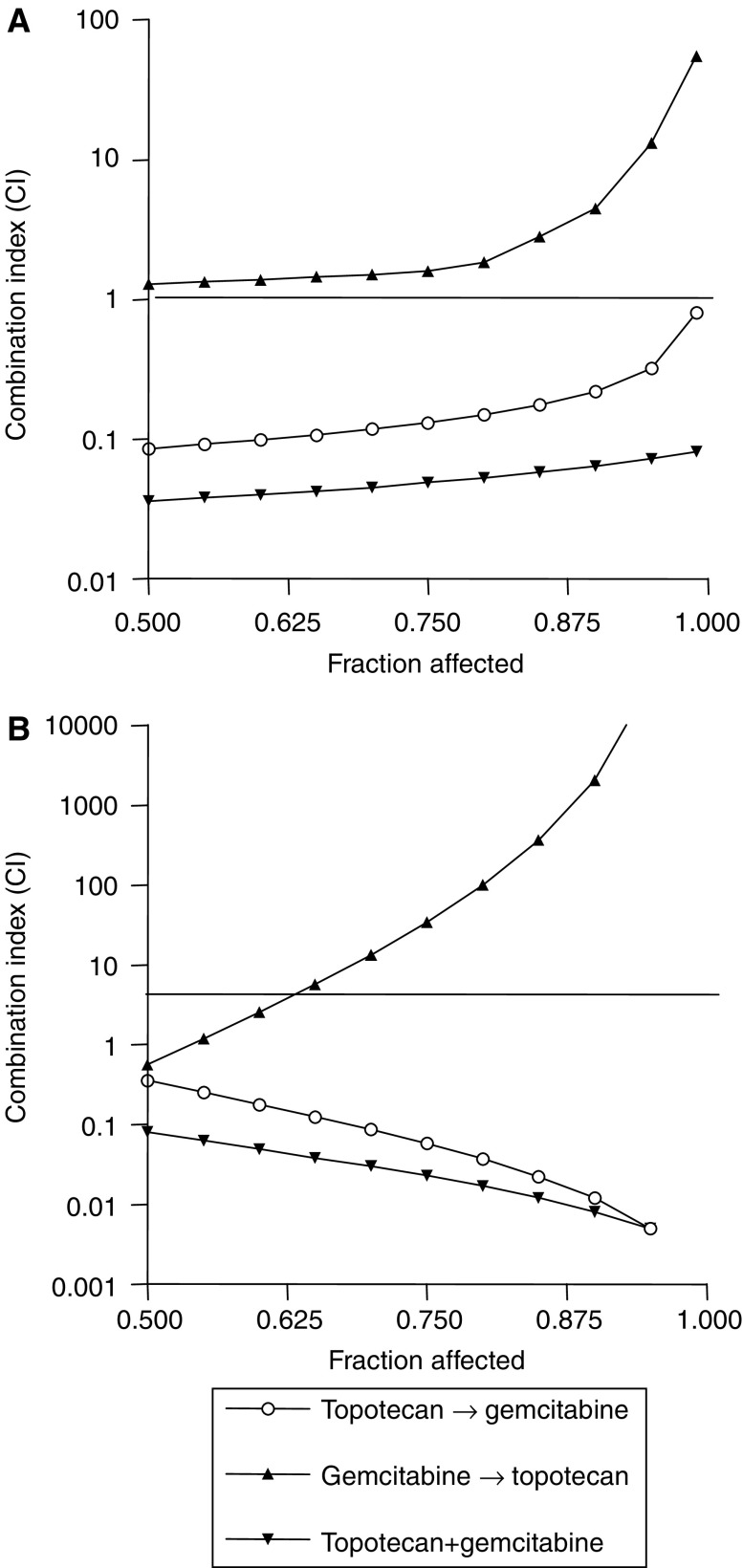
Combination index plots of topotecan–gemcitabine combination in A549 (**A**) and Calu-6 (**B**) cells. The most pronounced synergism (CI<1) was demonstrated when the two drugs were combined simultaneously in both cell lines.

A dose-dependent inhibition of cell growth was observed with gemcitabine and topotecan, with IC_50_ s of 131.2 and 840.2 ng ml^−1^ (A549 cells) and 1662.4 and 562.3 ng ml^−1^ (Calu-6 cells), respectively. Following on from this, because the CI method recommends a ratio of IC_50_ s values that the drugs are equipotent, combination studies were performed at fixed 6 : 1 and 1 : 3 (topotecan:gemcitabine) concentration ratios in A549 and Calu-6 cells, respectively. These studies showed that the sequential exposure of A549 cells to topotecan followed by gemcitabine reduced the IC_50_ of gemcitabine to 12.4 ng ml^−1^, while the IC_50_ of gemcitabine resulting from the simultaneous combination of drugs was 6.3 ng ml^−1^ (Table 1). The cytotoxic activity of gemcitabine was impaired when the drug was followed by topotecan (IC_50_, 620.1 ng ml^−1^). Similar results were obtained in Calu-6 cells, with IC_50_ s of 179.5, 40.4 and 2063.8 ng ml^−1^ after sequential topotecan → gemcitabine treatment, simultaneous combination and the gemcitabine → topotecan schedule. The calculation of the CI demonstrated that while the gemcitabine → topotecan sequential exposure showed antagonism at effect level >60%, the simultaneous and the topotecan → gemcitabine combination showed synergism at effect levels >50% inhibition, and the degree of synergism observed with the simultaneous administration of topotecan and gemcitabine was considerably greater in both cell lines (Figure 2).

### Cell cycle effects of gemcitabine and topotecan

**Figure 3:**
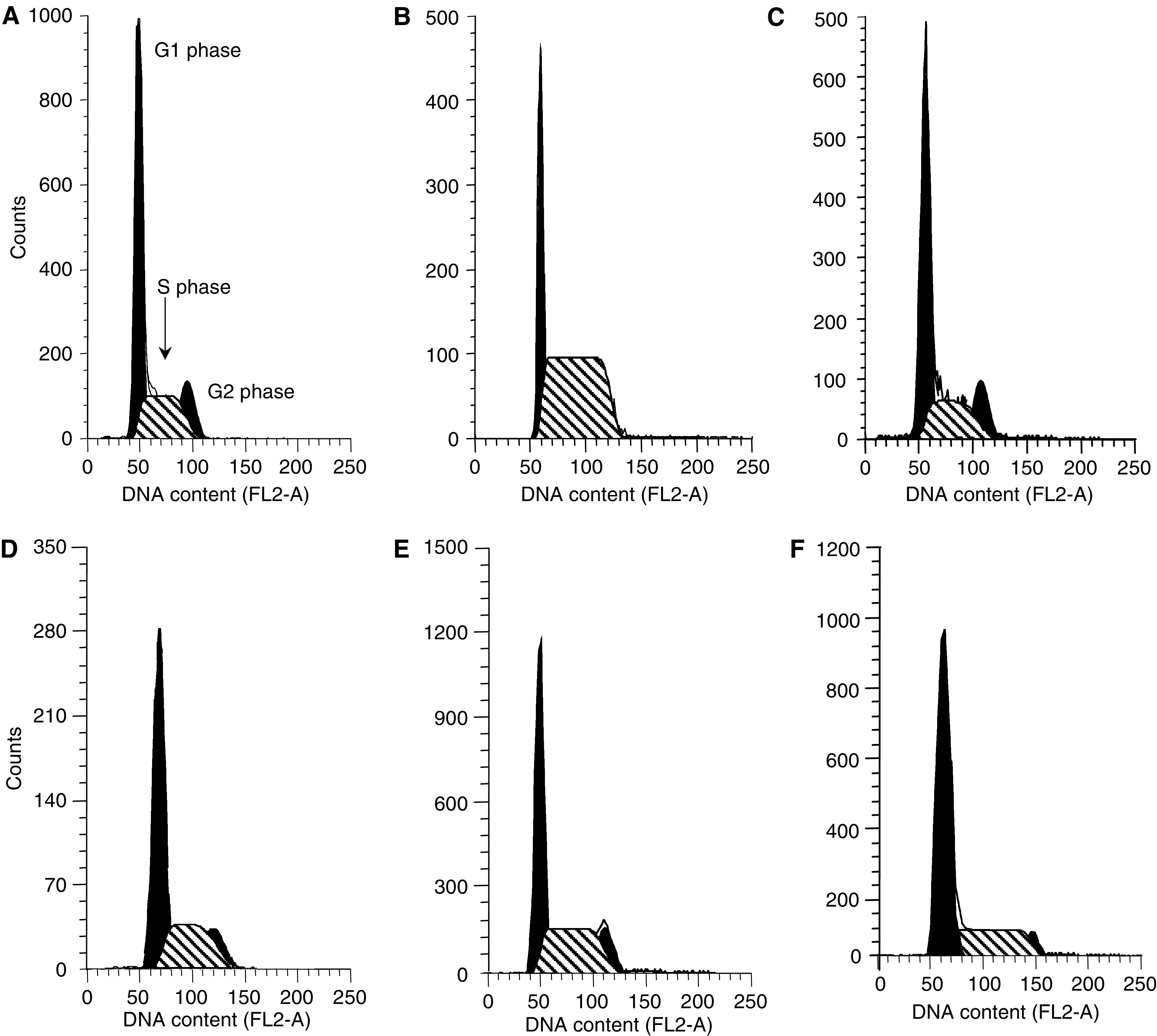
Histograms of DNA content of A549 (**A**–**C**) and Calu-6 (**D**–**F**) cells demonstrating the accumulation of cells in the S phase after topotecan treatment (**B** and **E**), and the minimal increase in the G1 phase after gemcitabine exposure (**C** and **F**) with respect to controls (**A** and **D**).

Both topotecan and gemcitabine were able to affect the cell cycle of lung cancer cells (Table 2). In particular, after 1 h treatment, topotecan markedly increased the percentage of cells in the S phase in A549 cells, from 39.6 to 64.9%, while there was a minimal enhancement (+6.1%) in Calu-6 cells. In contrast, flow cytometric studies demonstrated that gemcitabine blocked cells in the G1-S boundary. In particular, in Calu-6 cells, gemcitabine caused a 1.3-fold increase in the population of cells in the G1 phase, from 50.5 to 64.1% (Figure 3), while DNA contents of cells treated with gemcitabine showed a minimal increase (+5.8%) at the G1–S region in A549 cells (Figure 3).

### Induction of apoptosis by gemcitabine and topotecan

**Figure 4:**
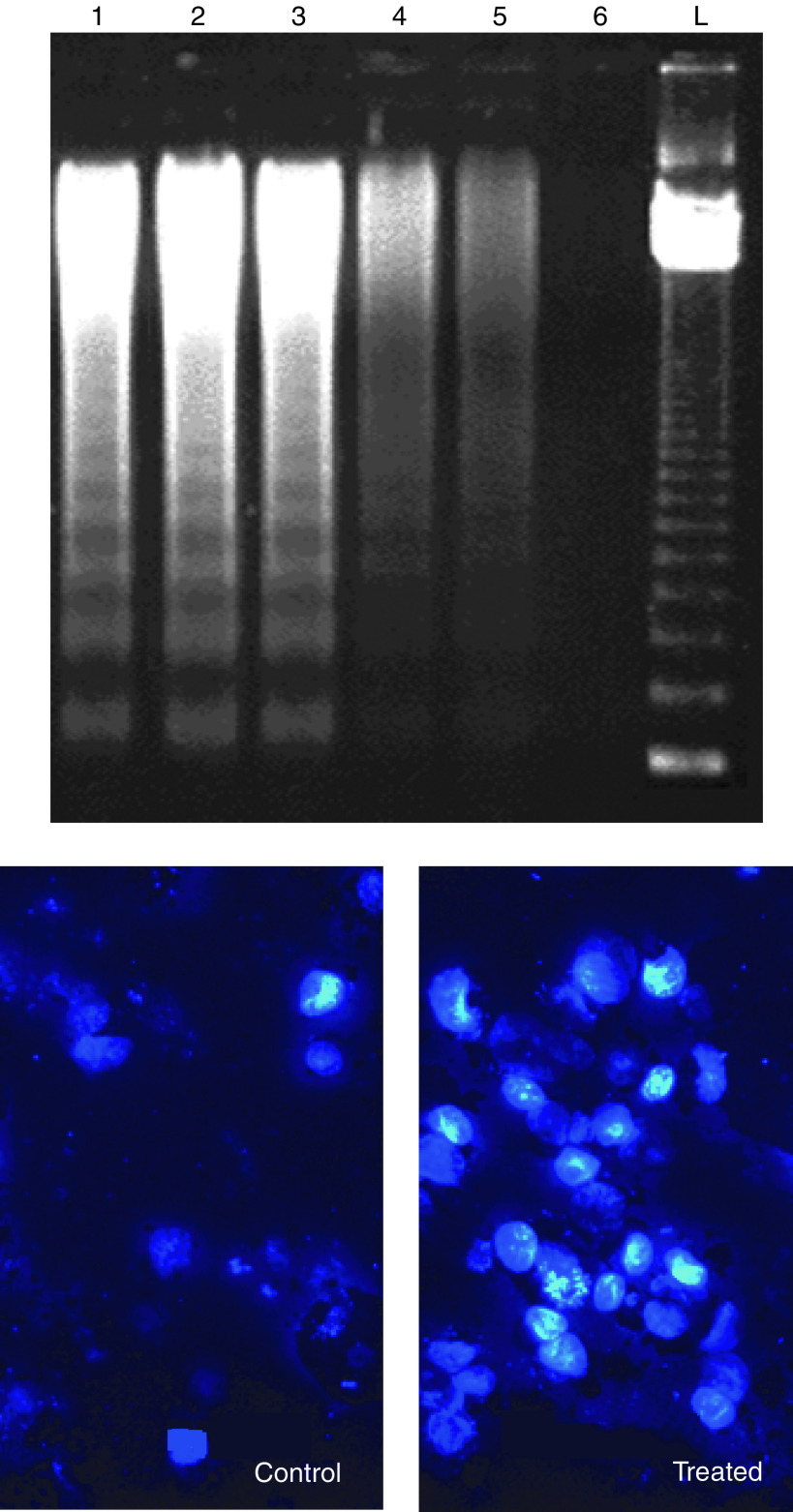
DNA fragmentation in A549 cells after treatment with gemcitabine (lane 4), gemcitabine and topotecan simultaneously (lane 1), topotecan followed by gemcitabine (lane 2), gemcitabine followed by topotecan (lane 3) and topotecan (lane 5) (above). The simultaneous combination of topotecan and gemcitabine at the 6 : 1 fixed concentration ratio determined the highest increase in apoptosis *vs* control (lane 6). L, 180 DNA ladder for fragment size identification. Nuclear staining of A549 cells exposed to gemcitabine–topotecan simultaneously. The typical apoptotic morphology includes nuclear condensation, cell shrinkage and formation of apoptotic bodies as compared to control cells (below).

The extent of DNA fragmentation was dependent on drug treatment. In particular, the production of chromatin fragments was clearly detectable after exposure to gemcitabine, topotecan and all the combinations in both cell lines, while no substantial amount of internucleosomal DNA fragmentation was observed in control samples (Figure 4). As shown in Figure 4, cells exposed to topotecan–gemcitabine combination presented typical apoptotic morphology with cell shrinkage, nuclear condensation and fragmentation, and rupture of cells into debris. Analysis of apoptosis by using bisbenzimide nuclear staining showed that the occurrence of apoptotic cells was significantly higher (*P*<0.05) after treatment with gemcitabine and topotecan (13.4±4.6 and 7.7±1.4%, respectively, *vs* controls, 4.3±0.6%, in A549 cells; and 8.6±2.0 and 6.1±0.4% *vs* 3.1±0.3% in Calu-6 cells). In the drug combinations, the simultaneous administration of gemcitabine and topotecan produced the highest apoptotic index either in A549 (34.0±3.6%) or in Calu-6 cells (27.6±3.6%), while the sequence topotecan → gemcitabine was more effective than the reverse sequence (Table 1).

### Activation of caspase-3 by gemcitabine and topotecan

Drug treatments were able to significantly increase the activity of caspase-3 over controls (0.12±0. 08 and 0.10±0.06 pmol min^−1^ mg^−1^ of protein in A549 and Calu-6 cells, respectively) (Table 1). In A549 cells, caspase-3 activities values were 0.86±0.14, 0.32±0.11 and 1.07±0.12 pmol min^−1^ mg^−1^ of protein, respectively, for gemcitabine, topotecan and the gemcitabine → topotecan sequence, while the simultaneous and the topotecan → gemcitabine sequence determined the maximum increase in caspase-3 activity, 2.76±0.10 and 1.43±0.11 pmol min^−1^ mg^−1^ of protein, respectively, *vs* untreated cells. Similar results were obtained in Calu-6 cells, with caspase-3 activities of 0.69±0.25, 0.39±0.13, 0.94±0.31, 2.55±0.37 and 1.55±0.28 pmol min^−1^ mg^−1^, after gemcitabine, topotecan, gemcitabine → topotecan, gemcitabine+topotecan or topotecan → gemcitabine treatments (Table 1).

### Inhibition of Akt phosphorylation

**Figure 5:**
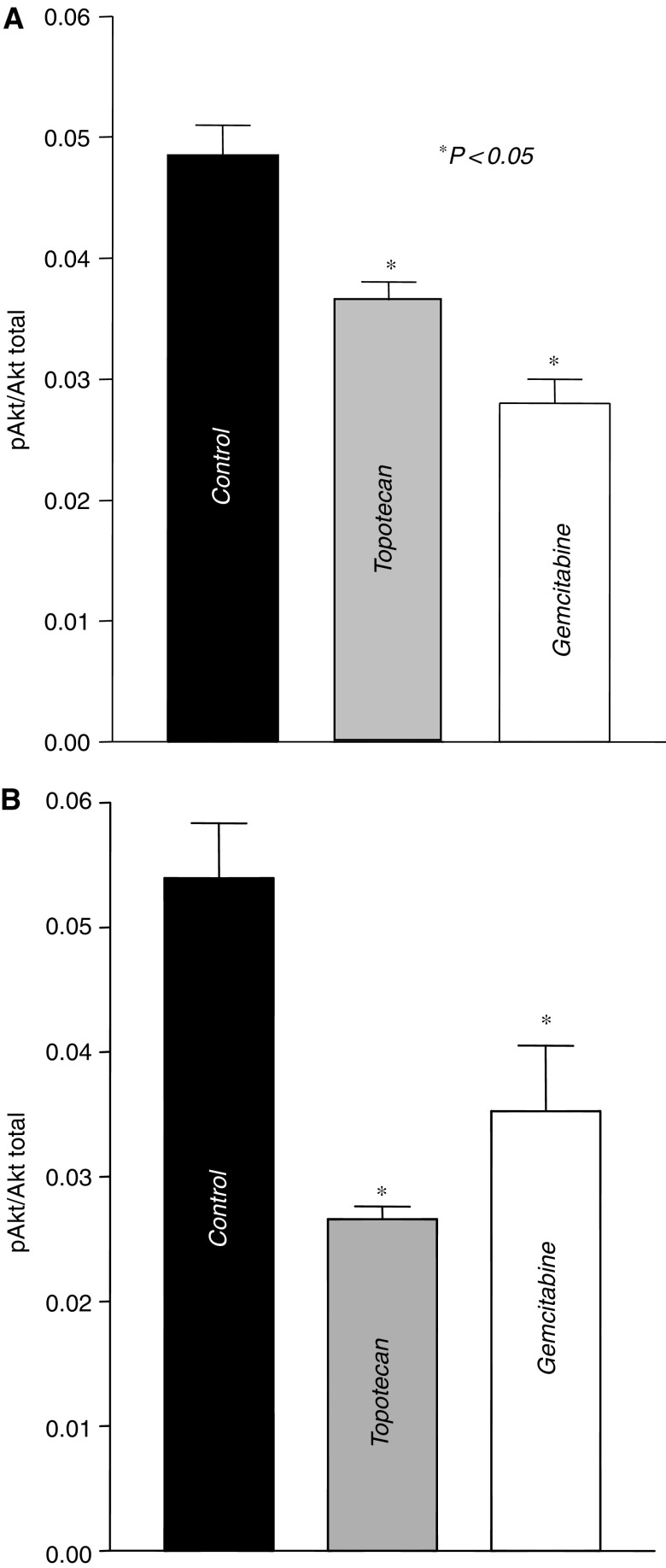
PI3-Akt-specific activity in drug-treated cells. The administration of gemcitabine and topotecan in A549 (**A**) and Calu-6 (**B**) cells determined a significant reduction of phosphorylated form (normalised to total Akt and protein content) *vs* control. Columns represent the data and bars the s.e. of values obtained from three independent experiments.

Both topotecan and gemcitabine were able to significantly reduce the amount of phosphorylated Akt in A549 and Calu-6 cells (*P*<0.05). Although drug treatments had no effect on total Akt protein expression, the amount of the phosphorylated form of Akt was decreased up to 27.1% (A549 cells) and 50.7% (Calu-6 cells) by topotecan and to 41.7% (A549 cells) and 34.7% (Calu-6 cells) by gemcitabine, in comparison with controls (Figure 5).

### Quantitative, real-time PCR analysis

**Figure 6:**
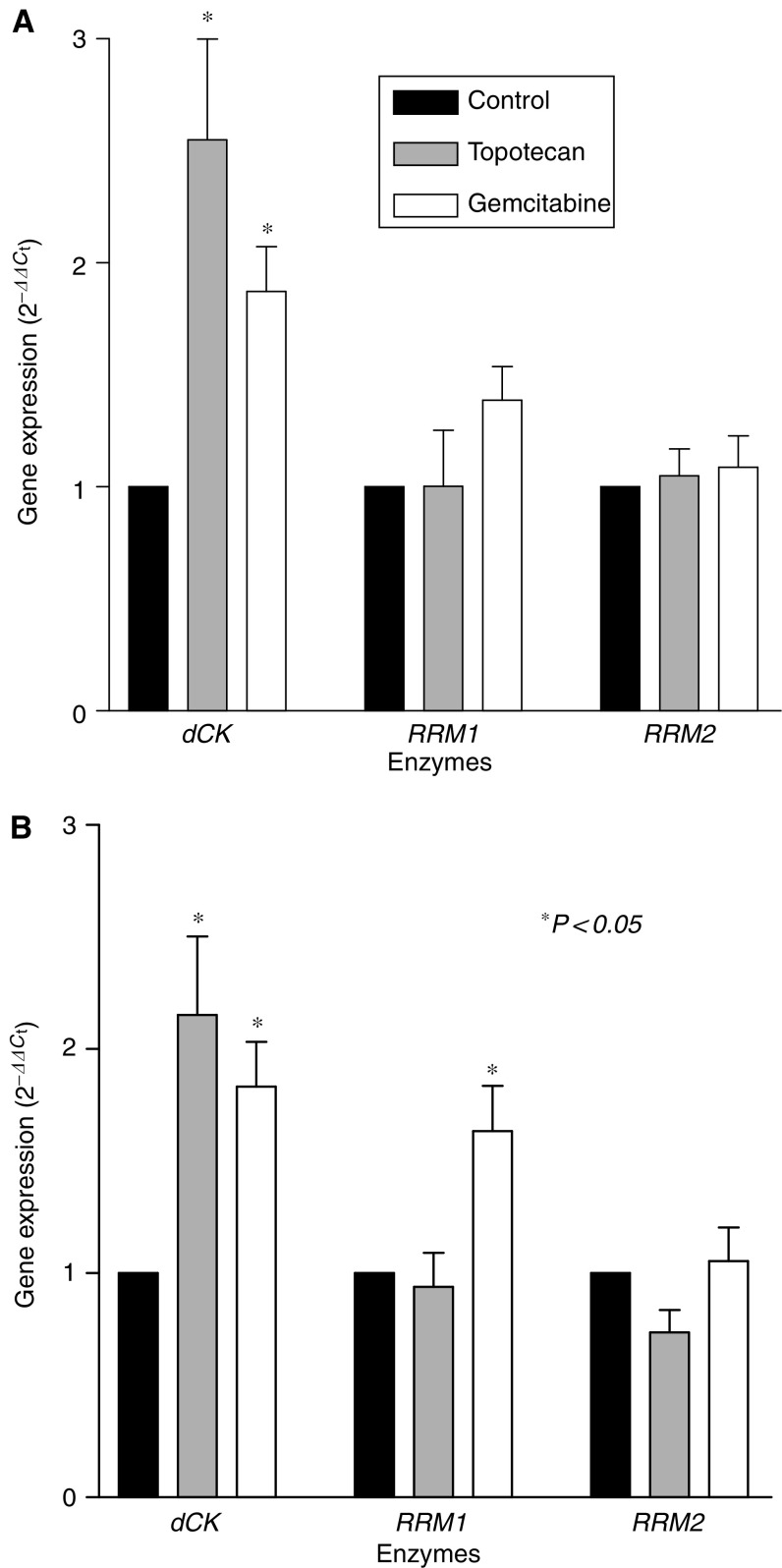
Expression of *dCK* and *RR* in the human NSCLC cell lines A549 (**A**) and Calu-6 (**B**). Values were calculated using the comparative ΔΔ*C*_t_ method, in which the amount of target, normalised to the endogenous control and relative to the calibrator (untreated control cells) is given as 2^-ΔΔ*C*_t_^. Columns represent the data and bars the s.e. of values from two independent experiments.

Quantitative, real-time PCR analysis was performed to assess whether the expression of *dCK* and *RR* was modulated by topotecan and gemcitabine, at IC_50_ levels. The results are given in Figure 6 and showed that both drugs significantly enhanced *dCK* expression, up to 155.0 and 115.3%, after topotecan treatment and 87.3 and 83.3% after gemcitabine exposure, respectively, in A549 and Calu-6 cells (*P*<0.05), while there was only a slight increase in *RRM1* expression (+38.7% in A549 and +63.5% in Calu-6 cells) after gemcitabine treatment.

## DISCUSSION

Several studies reported that gemcitabine showed synergism or additivity when combined with topotecan, cisplatin and etoposide in various NSCLC cell lines (Tolis *et al*, 1999; van Moorsel *et al*, 1999a, 1999b), while topotecan demonstrated synergism with etoposide in the NCI-H23 NSCLC cell line (Taron *et al*, 2000). The combination of gemcitabine and topotecan yielded conflicting results on various cancer cell lines. A recent study showed an additive cytotoxicity of gemcitabine followed by topotecan in H460 and H322 NSCLC cell lines (Tolis *et al*, 1999), while in A2780 ovarian cancer cells the same combination showed antagonism (Distefano *et al*, 2000). *In vitro* experimental findings reported in this study indicate that topotecan and gemcitabine administered simultaneously and in the sequence topotecan followed by gemcitabine were synergistic against A549 and Calu-6 cell lines, while the reverse sequence was antagonistic.

Recent studies have shown the importance of modulating the cell cycle to exploit the effect of drug combinations (Shah and Schwartz, 2001). In the present study, flow cytometry demonstrated that topotecan caused an accumulation of cells in the S phase. This finding is in agreement with previous data on increased proportion of NSCLC cells in S and G2/M phases after a 4-h exposure to topotecan (Tolis *et al*, 1999). As gemcitabine is a S-phase specific drug, the increase in its activity in the schedule topotecan → gemcitabine may be the result of modulation of cell cycle, potentially facilitating dFdCTP incorporation in DNA. Furthermore, the simultaneous use of gemcitabine and topotecan enhanced apoptosis, as demonstrated by the typical cellular morphology and the internucleosomal DNA fragmentation. There is substantial evidence that gemcitabine triggers apoptosis in human leukaemia and solid tumour cells, including NSCLC cell lines (Pace *et al*, 2000) and the concentration of gemcitabine required for this effect is similar to the serum levels achieved in clinical trials (Hui and Reitz, 1997). Triggering of the apoptotic machinery in response to a variety of cellular stresses and physiological stimuli culminates in the activation of caspases, resulting in cell death. Caspase-3 is the most widely studied enzyme with respect to the cleavage of target proteins (Fraser and Evan, 1996), and caspase-3-like protease is involved in the apoptotic death of A549 cells (Leech *et al*, 2000). Recent studies showed that treatment with gemcitabine triggers caspase-3 activity in A549 cells (Lawrence *et al*, 2001) and topoisomerase I is a substrate cleaved by caspase-3 (Samejima *et al*, 1999). Moreover, short exposures to camptothecin (e.g., 1 h) induce a dramatic decrease in topoisomerase I levels, resulting in a reduction of protein-linked DNA strand breaks. Therefore, the use of such agent simultaneously with other compounds, compared with the sequential administration of the same agents, would result in different cytotoxic effects (Fu *et al*, 1999). In agreement with these findings, *in vitro* experimental data obtained in this study indicate that gemcitabine significantly increase the activity of caspase-3 over control. Although a contradictory picture has emerged from previous studies on topoisomerase I degradation and loss of activity during apoptotic execution (Casiano *et al*, 1998), it might be possible that the caspase-3 activation caused by the initial gemcitabine exposure results in reduction of topoisomerase I, thus providing a possible explanation for the antagonism of the gemcitabine → topotecan sequence. On the other hand, in the reverse sequence and in the simultaneous drug treatment, the prevalence of proapoptotic mechanisms determined synergism. In particular, a recent study showed that the reduction of phosphorylated PKB/Akt levels correlated with the enhancement of gemcitabine-induced apoptosis and antitumour activity, suggesting that the PI3K-Akt pathway plays a significant role in mediating drug sensitivity in human cancer cells (Ng *et al*, 2000). In agreement with previous data on A549 cell line (Nakashio *et al*, 2000), this study demonstrated that topotecan decreased the amount of the activated form of Akt both in A549 and Calu-6 cells, so that it may improve the therapeutic potential of gemcitabine by increasing apoptosis when it is administered before or simultaneously.

The data of the present study also demonstrated for the first time that gemcitabine and, more effectively, topotecan increased the expression of *dCK*, potentially facilitating gemcitabine activation, while *RR* gene expression was not significantly modulated by topotecan in both cell lines. There are many observations suggesting that dCK is a limiting factor for the cytotoxic activity of gemcitabine. Several cell lines, which lack dCK activity, are resistant to nucleoside analogues such as cytarabine or gemcitabine (Bergman *et al*, 1999, 2002), and the sensitivity to nucleoside analoguess could be restored by transfection of a wild-type *dCK* cDNA (Stegmann *et al*, 1995). An increase in *dCK* expression was also observed after gemcitabine exposure in pancreatic cancer PANC-1 and Capan-1 cells (Giovannetti *et al*, 2004), suggesting that treatment with inhibitors of DNA synthesis could potentiate the *dCK* expression of various cells, because the salvage pathway initiated by dCK accounts for the majority of nucleotide synthesis for DNA repair. Other studies indicated that drugs that inhibit DNA biosynthesis widely differ in their stimulatory effect on dCK, and there are also ‘responsive’ and ‘nonresponsive’ cells with respect to dCK activation (Spasokoukotskaja *et al*, 1999). Thus, enhancement of the dCK activity by specific drugs in ‘responsive’ cells may give a rationale for combination chemotherapy.

Another hypothesis to explain the present results is that DNA repair is inhibited by topotecan, thereby resulting in increased gemcitabine incorporation into DNA. Indeed, Bergman *et al* (1996) reported that the synergism between gemcitabine and cisplatin appears to be mainly dependent on an increase in platinum-DNA adduct formation possibly related to changes in DNA caused by gemcitabine incorporation in DNA. Theoretically, the topoisomerase I-dependent strand breaks stabilised by topotecan offer sites for the insertion of gemcitabine during DNA duplication. Finally, a recent study showed that gemcitabine incorporation in cellular DNA enhanced the frequency of collisions between the stabilised topoisomerase I cleavage complex and transcription complexes, leading to accumulation of strand breaks and ultimately to enhanced cell death (Pourquier *et al*, 2002).

In conclusion, all these factors may contribute to the synergistic effect of topotecan and gemcitabine in NSCLC cell lines and provide the experimental basis for further clinical testing of these drug combination for the treatment of NSCLC.

## Figures and Tables

**Table 1:** Effects of gemcitabine, topotecan or their combinations on cell proliferation, caspase activity and apoptosis in A549 and Calu-6 cells. Data are the mean±s.e. from three independent experiments

**Treatment**	**Cells**	**IC_50_ (ng ml^−1^)^a^**	**Apoptotic index^b^**	**Caspase activity (pmol min^−1^ mg^−1^)^c^**
Control	A549	/	4.3±0.6%	0.12±0.08
	Calu-6	/	3.1±0.3%	0.10±0.06
				
Topotecan	A549	840.2±121.2	7.7±1.4%	0.32±0.11
	Calu-6	562.3±93.5	6.1±0.4%	0.39±0.13
				
Gemcitabine	A549	131.2±30.3	13.4±4.6%	0.86±0.14
	Calu-6	1662.4±360.6	8.6±2.0	0.69±0.25
				
Gemcitabine → topotecan	A549	620.1±104.1	10.5±0.7%	1.07±0.12
	Calu-6	2063.8±414.6	11.5±2.8%	0.94±0.31
				
Gemcitabine+topotecan	A549	12.4±1.4	34.0±3.6%	2.76±0.10
	Calu-6	40.4±4.76	27.6±3.6%	2.55±0.37
				
Topotecan → gemcitabine	A549	6.3±0.3	29.2±5.9%	1.43±0.11
	Calu-6	179.5±38.7	19.8±3.2%	1.55±0.28

a 50% inhibitory concentrations of cell growth.

b Percentage ratio between the number of cells displaying apoptotic appearance and the total number of counted cells.

c Enzyme activity is normalised with respect to total protein content of each cell extract.

**Table 2:** Cell cycle perturbation (%) induced by gemcitabine and topotecan. Data represent mean percentage from three independent experiments

**Cells**	**Treatment**	**G1 (%)**	**S (%)**	**G2 (%)**
A549	Control	46.06	39.58	14.36
	Gemcitabine	51.91	33.45	14.64
	Topotecan	33.42	64.89	1.69
				
Calu-6	Control	50.46	35.01	14.54
	Gemcitabine	64.10	31.62	4.27
	Topotecan	45.91	41.08	13.01
